# Translation, adaptation, and cross-cultural validation into Brazilian portuguese of the hearing protection assessment questionnaire (HPA)

**DOI:** 10.1590/2317-1782/20232021201en

**Published:** 2023-05-01

**Authors:** Luciana Bramati, Claudia Giglio de Oliveira Gonçalves, Jair Mendes Marques, Ravi Reddy, David Welch, Adriana Bender de Moreira Lacerda

**Affiliations:** 1 Programa de Mestrado e Doutorado em Distúrbios da Comunicação, Universidade Tuiuti do Paraná - UTP - Curitiba (PR), Brasil.; 2 Doctoral Program, School of Health Sciences, Massey University - Auckland, New Zealand.; 3 Department of Computer Schience, University of Auckland - Auckland, New Zealand.; 4 École d’orthophonie et d’audiologie, Université de Montréal - UdeM - Montréal, Canada.

**Keywords:** Hearing, Hearing Loss, Noise-Induced, Ear Protective Devices, Risk-Taking, Surveys and Questionnaires

## Abstract

**Purpose:**

The aim of the present study is to translate, adapt, and cross-culturally validate the Brazilian Portuguese version of the questionnaire Hearing Protection Assessment Questionnaire (HPA).

**Methods:**

The original instrument, developed in English, seeks to assess barriers and supports related to the use of hearing protection devices (HPD), as well as workers' knowledge, habits and attitudes towards occupational noise. The translation, adaptation, and cross-cultural validation of the questionnaire consisted of five steps: Translation of the questionnaire from English to Portuguese; 2) Reverse translation from Portuguese to English; 3) Analysis of the instrument by three experts in the field; 4) Pre-test of the questionnaire with ten workers; 5) Application of the instrument to 509 workers in a meatpacking industry after the pre-employment medical exam.

**Results:**

The results indicate the construction and content validity of the Brazilian Portuguese version for use with a working population and its internal consistency.

**Conclusion:**

This study resulted in the translation, cultural adaptation, and validation of the Hearing Protection Assessment Questionnaire (HPA), in order to be used to assess the use of individual hearing protection in the occupational field, called Hearing Protection Assessment Questionnaire (HPA).

## INTRODUCTION

The identification of knowledge, habits, and attitudes of workers regarding exposure to noise in the work environment is recommended to implement appropriate educational actions. This scenario suggests the need to use a specific instrument that performs this task.

The use of an instrument to assess the effectiveness of educational interventions aimed at workers on different dimensions or aspects related to occupational noise, hearing protection devices (HPD), and the prevention of hearing loss induced by high sound pressure levels (HLIHSPL) is an important resource to be used by occupational health and safety teams in the investigation process of exposure to high sound pressure levels. Thus, when the needs of individuals in the face of noise are known, we may be able to implement an educational process within the Hearing Preservation Program^(1)^.

Instruments for this purpose were used in national and international studies. Studies used multiple-choice questionnaires addressing the themes explored in educational interventions and applied pre- and post-intervention aiming to evaluate the effectiveness of these interventions regarding occupational noise^(2-7)^.

The questionnaire entitled “Beliefs and Attitudes about Hearing Protection,” originating from NIOSH (1996)^(8,9)^ in the United States, was translated, adapted, and validated for the Portuguese language in 2008^(10)^ and used in a study carried out in 2008^(11)^. This questionnaire consists of two parts (A and B) and assesses workers' beliefs and attitudes about preventing hearing loss and how they use HPD.

In a study carried out with firefighters, questionnaires focused on knowledge and attitudes towards HLIHSPL were sent by e-mail pre- and post-intervention. The results showed that educational intervention proved to be effective in increasing knowledge about HLIHSPL, as well as positive attitudes regarding the use of HPD, making its use more frequent among the study group (80% after the intervention and 20% before)^(12)^.

A study published in 2018 aimed to describe the knowledge of employees about the importance of using HPDs, the benefits of their use, and the harm caused by not using them constantly. The authors used a questionnaire prepared by them, applied before and after the intervention. The questionnaires contained 20 objective questions addressing the use of HPD, questions about continuous exposure to noise, and employees' knowledge about the type of equipment used in the company^(13)^.

Another study carried out in 2013^(14)^ sought to analyze the comfort of individual HPDs as part of an intervention to prevent hearing loss in workers exposed to high levels of noise through the use of a comfort assessment questionnaire.

Thus, the use of questionnaires as instruments for evaluating the effectiveness of educational intervention actions is extremely important and relevant, providing valuable subsidies for directing actions aiming hearing protection.

The educational intervention of the Dangerous Decibels program adapted for workers suggests the use of the instrument: Hearing Protection Assessment Questionnaire (HPA) developed by Reddy et al.^(15)^ before and after the intervention. This instrument assesses barriers and supports related to the use of individual HPD^(16)^, as well as workers' knowledge, habits, and attitudes towards occupational noise. As there is no version adapted to Brazilian Portuguese of the HPA that could be used in the intervention of the Dangerous Decibels Brasil (DDB) program for workers, this study presents the translation, adaptation, and cross-cultural validation of this instrument.

In the study by Reddy et al.^(15)^, the objective was to understand the personal and environmental factors that affect hearing protection behavior in workers and develop an intervention to promote it. The theoretical framework used for this study was the Ecological Model of Health Promotion. It is a planning model that helps to identify and target behavioral influences at various levels of the social environment through semi-structured interviews. The intervention used was the Dangerous Decibels program adapted for workers. The questionnaire Hearing Protection Assessment Questionnaire (HPA), used by Reddy et al.^(15)^, was a reliable and valid tool to identify the influences of hearing protection behavior at different levels^(17)^.

In this context, the objective of the present study is to translate, adapt, and cross-culturally validate the questionnaire Hearing Protection Assessment Questionnaire (HPA) developed by Reddy et al.^(15)^.

## METHODS

The present study was approved by the Ethics Committee of Universidade Tuiuti do Paraná, process no. 2,725,935, and approved by the company whose employees participated in this research. It should be noted that all individuals involved signed the Informed Consent.

### Instrument

The hearing protection assessment questionnaire that assesses five scales (HPA), developed and described by Reddy^(1)^, assesses barriers and supports, knowledge, attitudes, and behaviors in relation to HPD^(1,15)^.

Knowledge, attitudes, and behaviors were adapted from a questionnaire used to assess the effectiveness of the Dangerous Decibels^(17)^ program. The scales related to knowledge, attitudes, and behavior feature multiple-choice questions, each of which has only one correct answer. There are five questions for the knowledge scale on the science of sound, hearing loss and hearing conservation (questions 13 to 17), two questions related to measuring attitudes towards noise protection and hearing protection (questions 18 and 19), two questions about attitudes of safety behavior at work (questions 7 and 8), and three questions about behavior (questions 10, 20 and 21).

The questions related to barriers and supports describe the reasons why workers used (supports) or did not use (barriers) hearing protectors when exposed to noise at work. The two issues related to **Support** are the issues 9 and 11. Question 11 has four subscales in the responses (safety culture, risk justification, behavior motivation, and safety culture). The question related to **Barriers** is the question 12, with two subscales in the answers (risk justification and restrictions on HPD use).

The questionnaire also includes demographic items such as gender and age (questions 1 to 6), an item to identify the self-reported frequency of individual HPD use (question 22), and an item to identify the self-reported frequency of co-workers' hearing protection behavior (question 23).

The questionnaire must be analyzed by comparing the answers to each question of the five dimensions separately (attitude, behavior, knowledge, supports, and barriers) before and after the educational intervention to detect differences in results between the two moments.

As the five dimensions evaluated in the pre- and post-educational intervention questionnaire have different numbers of items, the scores of correct answers must be converted into percentages to allow comparability between them.

### Translation, adaptation, and cross-cultural validation of the instrument

The process of translation, adaptation, and cross-cultural validation consisted of five steps in accordance with the recommendations of the WHO^(18)^ and COSMIN^(19)^. The researcher's participation in the adaptation of an instrument is desirable, since it allows quoting the concepts explored, reformulating the questions, and avoiding locutions and idiomatic expressions^(20)^.

*The first step* was the translation of the questionnaire from English into Portuguese, carried out by a bilingual teacher and revised by two experts in the field. At this stage, the semantic equivalence (grammar and vocabulary) and the cultural equivalence of each item (experiences lived within the cultural context of society) were evaluated.

*The second step* was to write the final version with the adjustments made by an expert in the area, and then forward it to a second expert (without any contact or information about the original version), so that he could proceed with the process translation from Portuguese into English. This translation was revised and compared with the original version by three experts (bilingual) to verify if there was any mischaracterization of the questionnaire.

*In the third stage*, the questionnaire was sent to three experts in the area, along with an instrument for them to express their comments on the translation performed.

This method seeks to facilitate understanding and make the instrument applicable to Brazilian Portuguese while maintaining equivalence between the original and the translation.

*In the fourth stage*, ten workers were selected (randomly) from another company to take part in the pre-test of the questionnaire at a time that did not interfere with their work activities.

The pre-test questionnaires were applied by a researcher at a predetermined time and, after reading and explanation by the applicator (about the research objectives and how the answers should be given), the workers were asked to answer the questionnaire, record the difficulties of interpretation, give their opinion on the language used (if it was adequate and/or there was an unknown word or expression), and indicate the difficulties encountered in answering it. Specific care with filling instructions and consistency of presentation were also evaluated. At this stage, no change in the questionnaire was necessary, as the workers did not have difficulties in interpreting and approved the language used.

*In the fifth stage*, a Work Safety Technician applied the questionnaire, responsible for integration groups (time of admission of the worker to the company). Workers of a meatpacking industry in the municipality of Chapecó, state of Santa Catarina, Brazil, participated in this stage. As an inclusion criterion, literate workers, over 18 years old, of both sexes, who were admitted to the company from September/2018 to March/2019, participated. Exclusion criteria comprised workers admitted in the same period, who did not know how to read or write, who did not speak Portuguese, and those under 18 years of age.

### Statistical analysis

For the analysis of content validity, the CVI (content validity index) was used, which is calculated based on the evaluations of the judges (experts)^(8)^. The CVI assesses the proportion or percentage of expert agreement on certain aspects of an instrument and its items^(21)^.

The reliability of the translated instrument was performed using the split-half method, and the sample was divided into two groups: one with 254 employees and the other with 255 employees. One was the upper half and the other was the lower half. Then, we compared the results for each question through the modified C Contingency Coefficient in the case of nominal questions, thus verifying their significance. The significance level of p<0.05 (5%) was adopted.

## RESULTS

### Results from the first to the fourth stage

Chart 1 shows the original version of the questionnaire, the process of translation, back translation, and adaptation of the questions and the answer options.

**Chart 1:** Original version of the questionnaire, the process of translation, back translation, and adaptation of the questions and the answer options

SCALE	ORIGINAL ENGLISH VERSION		TRANSLATION INTO BRAZILIAN PORTUGUESE		BACK-TRANSLATION INTO ENGLISH		SPECIALISTS COMMITTEE: SEMANTIC, LANGUAGE, CULTURAL, AND LINGUISTIC EQUIVALENCE	
Attitude	7. Please read the two statements carefully and choose the one which is most true for you: Please choose either A or B		7. Por favor, leia as duas frases cuidadosamente e escolha aquela que é mais verdadeira para você: (escolha A ou B)		7. Please read the two statements carefully and choose the one that is most true to you: (please choose A or B)		7. Por favor, leia as duas frases cuidadosamente e escolha aquela que é mais verdadeira para você: Por favor, escolha A ou B	
	A. Safety is at the forefront of my mind when working		A. Segurança está em primeiro lugar em minha mente quando trabalho		A. Safety comes first in my mind when I work		A. Para mim, a segurança está em primeiro lugar quando eu trabalho.	
	B. Safety is important, but other factors sometimes limit my ability to work safely		B. Segurança é importante, mas outros fatores às vezes limitam minha habilidade para trabalhar de forma segura		B. Security is important, however other factors sometimes limit my ability to work safely		B. Para mim, a segurança é importante, mas outros fatores ou condições de trabalho, às vezes limitam à minha maneira de trabalhar de forma segura.	
Attitude	8. Please read the two statements carefully and choose the one which is most true for you: Please choose either A or B		8. Por favor, leia as duas frases cuidadosamente e escolha aquela que é mais verdadeira para você: (escolha A ou B)		8. Please read the two statements carefully and choose the one that is most true to you: (please choose A or B)		8. Por favor, leia as duas frases cuidadosamente e escolha aquela que é mais verdadeira para você:	
	A. Injuries occur at work because people don’t take enough interest in safety		A. Os danos ocorrem no trabalho porque as pessoas não têm interesse suficiente em segurança		A. Damage occurs at work because people do not have enough interest in safety		A. Os danos ocorrem no trabalho porque as pessoas não têm interesse suficiente em segurança	
	B. Injuries at work will always occur, no matter how hard people try to prevent them		B. Os danos no trabalho sempre ocorrerão, não importa o quanto as pessoas tentem preveni-los(as)		B. Damage at work will always occur, no matter how much people try to prevent it.		B. Os danos sempre ocorrerão no trabalho, não importa o quanto as pessoas tentem preveni-los(as)	
Support	9. I have earplugs and or earmuffs to use at work		9. Eu tenho protetores auditivos para usar no meu trabalho		9. I have hearing protectors to use in my work		9. Eu recebo protetores auditivos para usar no meu trabalho	
	□ Yes	□ No	Sim □	Não □	□ Yes	□ No	Sim □	Não □
Behavior	10. I wear earplugs and or earmuffs when it is noisy at work (please circle one)		10. Eu uso só protetores auditivos quando tem barulho no meu ambiente de trabalho (por favor, assinale uma das opções abaixo)		10. I wear hearing protectors when it is noisy at work (please check one of the options below)		10. Eu uso protetores auditivos quando tem ruído no trabalho (por favor, assinale uma das opções abaixo)	
	□ Always		Sempre □		□ Always		Sempre □	
	□ Almost Always		Quase sempre □		□ Almost Always		Quase sempre □	
	□ Usually		Geralmente □		□ Usually		Geralmente □	
	□ Often		Muitas vezes □		□ Often		Muitas vezes □	
	□ Sometimes		Às vezes □		□ Sometimes		Às vezes □	
	□ Rarely or Never		Raramente ou Nunca □		□ Rarely or Never		Raramente ou Nunca □	
Support/ subscales	11. If you wear earmuffs or earplugs when exposed to noise, it is because: (please tick all those that apply)		11. Se você usa protetores auditivos no trabalho, é porque: (marque todas aquelas que se aplicam)		11. If you wear hearing protectors at work, it is because: (check all that apply)		11. Se você usa protetores auditivos no trabalho, é porque: (por favor, marque todas aquelas que se aplicam)	
Safety culture	A. Your boss tells you to		A. Seu chefe diz para você fazer		A. Your boss tells you to do		A. Seu chefe diz para você usar	
Hazard recognition	B. You are doing a noisy job (e.g., working on noisy machine, banging, hammering, etc.)		B. Você está fazendo um trabalho barulhento (ex.: trabalhando em máquinas ruidosas, com estrondos, pancadas, marteladas, etc.)		B. You are doing a noisy work (e.g., working on noisy machines, with bangs, banging, hammering, etc.)		B. Você está fazendo um trabalho ruidoso (ex.: trabalhando em máquinas ruidosas, com estrondos, pancadas, marteladas, etc.)	
Hazard recognition	C. Other workers are doing noisy jobs (e.g., working on noisy machine, banging, hammering, etc.)		C. Outros trabalhadores estão executando tarefas barulhentas (ex.: trabalhando em máquinas ruidosas, com estrondos, pancadas, marteladas, etc.)		C. Other workers are performing noisy tasks (e.g., working on noisy machines, with bangs, bangs, hammering, etc.)		C. Outros trabalhadores estão fazendo tarefas ruidosas (ex.: trabalhando em máquinas ruidosas, com estrondos, pancadas, marteladas, etc.)	
Behavior motivation	D. You want to protect your hearing		D. Você quer proteger sua audição		D. You want to protect your hearing		D. Você quer proteger sua audição	
Behavior motivation	E. You are annoyed by the noise		E. Você fica chateado com o barulho		E. You are upset / annoyed by the noise		E. Você fica incomodado com o ruído	
Behavior motivation	F. You want your hearing to be good to live a good life with your family		F. Você quer que sua audição esteja boa para viver uma vida com qualidade com sua família		F. You want a good hearing to have a good live and quality of life with your family		F. Você quer que sua audição esteja preservada para viver com qualidade junto à sua família	
Behavior motivation	G. Your workmates remind you to wear them		G. Seus colegas de trabalho te lembram de usá-los		G. Your co-workers remind you to use them		G. Seus colegas de trabalho te lembram de usá-los	
Safety culture	H. It is your company rules		H. São regras de sua empesa		H. It is your Company rules		H. São regras da sua empresa	
Safety culture	I. You have received training to wear them		I. Você recebeu treinamento para usá-los		I. You have received training to use them		I. Você recebeu treinamento para usá-los	
	J. Other, please specify:		J. Outro, por favor, especifique:		J. Other, please specify:		J. Outro, por favor, especifique: _______________________________	
Barriers/ subscales	12. If you don’t wear earmuffs or earplugs when exposed to noise, it is because: (please tick all those that apply)		12. Se você não usa protetores auditivos quando está exposto ao barulho, é porque: (marque todas aquelas que se aplicam)		12. If you don’t use hearing protectors when exposed to noise, it is because: (check all that apply)		12. Se você não usa protetores auditivos quando está exposto ao ruído, é porque: (por favor, marque todas aquelas que se aplicam)	
Risk justification	A. You are not clear as to when you should wear them		A. Não está claro para você quando você deveria usá-los		A. It is not clear to you when you should use them		A. Não está claro para você quando você deveria usá-los	
Hpd constraints	B. You can’t hear properly to do your work (e.g., warning signals, machine performance)		B. Você não consegue ouvir apropriadamente para fazer seu trabalho (ex. Sinais de aviso, performance de máquinas)		B. You cannot hear properly to do your job (e.g., warning signs, machine performance)		B. Você não consegue ouvir adequadamente para fazer seu trabalho (ex. Sinais de aviso, performance de máquinas)	
Hpd constraints	C. You can’t communicate properly with other workers		C. Você não consegue se comunicar apropriadamente com outros trabalhadores		C. You can’t to communicate properly with other workers		C. Você não consegue se comunicar adequadamente com outros trabalhadores	
Hpd constraints	D. They are uncomfortable		D. Eles são desconfortáveis		D. They are uncomfortable		D. Eles são desconfortáveis	
Hpd constraints	E. They get in the way of other safety equipment		E. Eles atrapalham o uso de outros equipamentos de segurança		E. They get in the use of other safety equipment		E. Eles atrapalham o uso de outros equipamentos de segurança	
Risk justification	F. You are used to noise at work		F. Você está acostumado com barulho no trabalho		F. You just used to noise at work		F. Você está acostumado com o ruído no trabalho	
Risk justification	G. Your co‐workers often don’t wear them		G. Seus colegas frequentemente não o usam		G. Your colleagues often don't use it		G. Seus colegas frequentemente não o usam	
Risk justification	H. Your co‐workers find it funny when you wear them		H. Seus colegas acham engraçado quando você os usa		H. Your colleagues find it funny when you use them		H. Seus colegas acham engraçado quando você os usa	
Risk justification	I. Someone else does something noisy without warning		I. Outras pessoas também fazem tarefas barulhentas sem aviso		I. Someone else does something noisy tasks without warning		I. Outras pessoas também fazem tarefas ruidosas sem avisar	
	J. Other, please specify:		J. Outros, por favor, especifique:		J. Other, please specify:		J. Outros, por favor, especifique:	
Knowledge	13. Hearing loss can be cured by getting hearing aids. (tick only one)		13. A Perda auditiva pode ser curada por aparelhos auditivos (Selecione apenas um)		13. Hearing loss can be cured by hearing aids (Select only one)		13. Perda auditiva pode ser curada com o uso de aparelhos auditivos (Selecione apenas um)	
	□ True		Verdadeiro □		□ True		Verdadeiro □	
	□ False		Falso □		□ False		Falso □	
	□ Not sure		Não tenho certeza □		□ Not sure		Não tenho certeza □	
Knowledge	14. Sounds measuring _______ and over are damaging to human hearing. (tick only one)		14. Medidas de som de _______________ e acima podem prejudicar a audição humana. (selecione apenas uma)		14. Sound measurements of _______________ and over are damaging to human hearing. (select only one)		14. Medidas de som de _______________ e acima podem prejudicar a audição humana. (selecione apenas uma)	
	65 decibels (dBA) □		65 decibels (dBA) □		65 decibels (dBA) □		65 decibels (dBA) □	
	70 decibels (dBA) □		70 decibels (dBA) □		70 decibels (dBA) □		70 decibels (dBA) □	
	85 decibels (dBA) □		85 decibels (dBA) □		85 decibels (dBA) □		85 decibels (dBA) □	
	Not sure □		Nenhuma destas alternativas □		None of these alternatives □		Nenhuma destas alternativas □	
Knowledge	15. Sounds that are too loud can damage the _______, causing hearing loss. (tick only one)		15. Os sons muito altos podem prejudicar __________________, causando perda auditiva (selecione uma apenas)		15. Sounds that are too loud can damage the __________________, causing hearing loss (select only one)		15. Sons muito altos podem prejudicar __________________, causando perda auditiva (assinale a alternativa que melhor preenche a frase acima e selecione uma apenas)	
							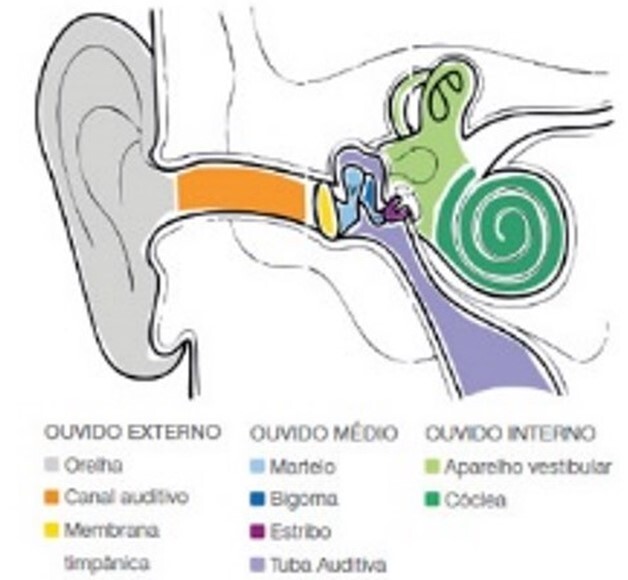	
	Ear drum □		Tímpano □		Ear drum □		Canal Auditivo □	
	Ear canal □		Canal Auditivo □		Ear canal □		Tímpano ou membrana timpanica □	
	Hair cells of the inner ear □		Células ciliadas do Ouvido Interno □		Hair cells of the inner ear □		Células ciliadas da cóclea □	
	All of the above □		Todas as opções acima □		All of the above □		Todas as opções acima □	
Knowledge	16. Hearing loss caused by loud sounds is something people ________ may have. (tick only one)		16. A perda auditiva causada por sons altos é algo que pessoas ___________________ podem ter (selecione apenas uma)		16. Hearing loss caused by loud sounds is something people ___________________ may have (select only one)		16. Perda auditiva causada por sons altos/ruídos é algo que pessoas ___________________ podem ter (assinale a alternativa que melhor preenche a frase acima e selecione apenas uma)	
	Over age 60 □		Acima dos 60 anos □		Over age 60 □		Acima dos 60 anos □	
	Over age 50 □		Acima dos 50 anos □		Over age 50 □		Acima dos 50 anos □	
	Over age 40 □		Acima dos 40 anos □		Over age 40 □		Acima dos 40 anos □	
	At any age □		Em qualquer idade □		At any age □		Em qualquer idade □	
Knowledge	17. I can protect my hearing from noise at work by wearing earmuffs or earplugs (tick only one)		17. Posso proteger minha audição de barulhos no trabalho usando protetores auditivos. (selecione apenas uma)		17. I can protect my hearing from noise at work by using hearing protection. (select only one)		17. Posso proteger a minha audição do ruido no trabalho usando protetores auditivos. (selecione apenas uma)	
	All the time when it is noisy □		Todo momento quando está barulhento □		All the time when it is noisy □		A Todo momento quando está ruidoso □	
	Only when I am doing a noisy job □		Apenas quando outras pessoas estão usando os seus □		Only when I am doing a noisy job □		Apenas quando outras pessoas estão usando os seus □	
	Only when others are wearing theirs □		Apenas quando o barulho me incomoda □		Only when others are wearing theirs □		Apenas quando o ruído me incomoda □	
	Only when my boss tells me to □		Apenas quando estou fazendo um trabalho barulhento □		Only when my boss tells me to □		Apenas quando estou fazendo um trabalho ruidoso □	
	Only when the noise annoys me □		Apenas quando meu chefe me orienta a usar □		Only when the noise annoys me □		Apenas quando meu chefe me orienta a usar □	
Attitude	18. Having a hearing loss is not a big deal (tick only one)		18. Ter perda auditiva não é um grande problema (selecione apenas uma)		18. Having hearing loss is not a big problem (select only one)		18. Adquirir uma perda auditiva não é um grande problema para mim (selecione apenas uma)	
	Agree □		Concordo □		Agree □		Concordo □	
	Disagree □		Discordo □		Disagree □		Discordo □	
	Not sure □		Não tenho certeza □		I'm not sure □		Não tenho certeza □	
Attitude	19. Workers who listen to loud sounds all the time don't seem to have a hearing loss, so I don't have to worry about getting a hearing loss. (tick only one)		19. Trabalhadores que escutam sons altos o tempo todo não parecem ter perda auditiva, assim, eu não tenho que me preocupar se tiver perda auditiva (selecione uma apenas)		19. Workers who listen loud sounds all the time don't seem to have a hearing loss, so I don't have to worry about getting a hearing loss (select only one)		19. Trabalhadores que estão expostos a ruídos o tempo todo não parecem ter perda auditiva, assim, eu não tenho que me preocupar se tiver perda auditiva (selecione uma apenas)	
	Agree □		Concordo □		Agree □		Concordo □	
	Disagree □		Discordo □		Disagree □		Discordo □	
	Not sure □		Não tenho certeza □		I'm not sure □		Não tenho certeza □	
Behavior	20. If it is noisy, and my workmates are not wearing earmuffs or earplugs. (tick only one)		20. Se estiver barulhento, e meus colegas de trabalho não estiverem usando protetores auditivos (selecione uma apenas)		20. If it is noisy, and my co-workers are not wearing hearing protection (select only one)		20. Se o ambiente de trabalho estiver ruidoso, e meus colegas de trabalho não estiverem usando protetores auditivos (selecione uma apenas)	
	I carry on with my work and let them do what they want □		Eu prossigo com meu trabalho e os deixo fazerem o que eles quiserem □		I carry on with my work and let them do what they want □		Eu prossigo com meu trabalho e os deixo fazerem o que eles quiserem □	
	I remind and encourage them to wear their earplugs or earmuffs □		Eu lembro e incentivo meus colegas a usarem seus protetores auditivos □		I remind and encourage my colleagues to wear their hearing protectors □		Eu lembro e incentivo meus colegas a usarem seus protetores auditivos □	
	I also take mine off because they are not wearing theirs □		Eu também tiro os meus porque eles não estão usando os deles □		I also take mine because they are not using theirs □		Eu também tiro os meus porque eles não estão usando os deles □	
Behavior	21. During the past week, I have been around dangerously loud sounds at work without wearing hearing protection. (tick only one)		21. Durante a semana passada, eu estive em contato com sons altos no trabalho sem usar proteção auditiva. (selecione uma apenas)		21. During the past week, I have been around dangerously loud sounds at work without wearing hearing protection. (select one only)		**21.** Durante a semana passada, eu estive exposto a ruídos no trabalho sem usar proteção auditiva. (selecione uma apenas)	
	Yes □	No □	Sim □	Não □	Yes □	No □	Sim □	Não □
Behavior	22. I wear earplugs or earmuffs when other workers are doing a noisy job at work (tick only one)		22. Uso protetores auditivos quando outros trabalhadores estão fazendo um serviço barulhento no trabalho.		22. I use hearing protection when other workers are doing a noisy job at work.		22. Eu uso protetores auditivos quando outros trabalhadores estão fazendo um serviço ruidoso no trabalho.	
	Always □		Sempre □		Always □		Sempre □	
	Almost always □		Quase sempre □		Almost always □		Quase sempre □	
	Usually □		Geralmente □		Usually □		Geralmente □	
	Often sometimes □		Frequentemente □		Often sometimes □		Frequentemente □	
	Rarely □		Raramente □		Rarely □		Raramente □	
	Never □		Nunca □		Never □		Nunca □	
Behavior	23. How often do your workmates wear earmuffs or earplugs when it is noisy? (tick only one)		23. Com que frequência seus colegas de trabalho usam protetores auditivos quando está barulhento? (Selecione uma apenas)		23. How often do your co-workers wear hearing protection when it is noisy? (Select one only)		23. Com que frequência seus colegas de trabalho usam protetores auditivos quando o ambiente de trabalho está ruidoso? (Selecione uma apenas)	
	Always □		Sempre □		Always □		Sempre □	
	Almost always □		Quase sempre □		Almost always □		Quase sempre □	
	Usually □		Geralmente □		Usually □		Geralmente □	
	Often sometimes □		Às vezes □		Often sometimes □		Às vezes □	
	Rarely □		Raramente □		Rarely □		Raramente □	
	Never □		Nunca □		Never □		Nunca □	

The team of experts that analyzed the translations (third stage) pointed out that there was correspondence in the translated items, semantic equivalence between the two translations for most questions, and absence of translation difficulties. Adjustments were made for differences in verbal agreement. The counter-translation with the original version did not reveal a need for changes in grammatical structures when the Portuguese version was translated into English.

The expert committee's judgment reveals that questions 8, 9, 10, 13, 14, and 16 reached consensus among the three judges. Questions 11 (B/C/E), 12 (F/I), 17, 19, 20, 22, and 23 were considered items in need of minor revisions to be representative, and questions 7, 15, 18, and 21 underwent changes that aimed to facilitate understanding in the Portuguese language considering the cultural differences between languages.

The Content Validity Index (CVI) evaluated the proportion of expert agreement on the instrument and its items. Table 1 shows the proportion of questions that were scored by the judges (experts).

**Table 1:** Content Validity Index (CVI)

QUESTION	CVI		QUESTION	CVI	QUESTION	CVI
Q7	0.33		Q13	1.00	Q19	1.00
Q8	1.00		Q14	1.00	Q20	1.00
Q9	1.00		Q15	0.67	Q21	0.67
Q10	1.00		Q16	1.00	Q22	1.00
Q11	1.00		Q17	1.00	Q23	1.00
Q12	1.00		Q18	0.67		

Table 1 shows that the questions with CVI = 1.00 received scores 3 or 4 among the three judges, therefore with adequate content validity. Questions with CVI = 0.33 or CVI = 0.67 were those that received at least a score of 1 or 2, therefore these questions were revised. Table 1 presents the Content Validity Index (CVI).

In the fourth stage, the ten workers answered the questionnaire (Chart 2). They did not present difficulties in interpretating questions and considered the language adequate. After answering the questionnaire, some asked about comfortable noise levels and types of hearing protectors used.

**Chart 2:** Final Brazilian Portuguese version of the questionnaire: **Hearing Protection Assessment (HPA)**

Como responder: preencha a sua resposta com um **X** ou escreva as suas respostas nas linhas.

**1. Gênero:** Masculino □ Feminino □
**2. Idade:** _____________anos Data de Nascimento: _____/_____/_____
**3. Cargo:** _______________________________ Turno: ________________
**4. A qual grupo étnico você pertence?** __________________________________

**5. Qual é seu país de nascimento?**
Brasil □ outro, por favor, especifique: _____________________________
**6. Se você respondeu « outro » para a questão 5, há quantos anos você está no Brasil?** ______
**7. Por favor, leia as duas frases cuidadosamente e escolha aquela que é mais verdadeira para você. Por favor, escolha A ou B:**
A. Para mim, a segurança está em primeiro lugar quando eu trabalho.
B. Para mim, a segurança é importante, mas outros fatores ou condições de trabalho, às vezes limitam à minha maneira de trabalhar de forma segura.
**8. Por favor, leia as duas frases cuidadosamente e escolha aquela que é mais verdadeira para você. Por favor, escolha A ou B:**
A. Os danos ocorrem no trabalho porque as pessoas não têm interesse suficiente em segurança
B. Os danos sempre ocorrerão no trabalho, não importa o quanto as pessoas tentem preveni-los
**9. Eu recebo protetores auditivos disponíveis para usar no meu trabalho.**
Sim □ Não □
**10. Eu uso protetores auditivos quando tem ruído no trabalho (por favor, assinale uma das opções abaixo):**
Sempre □ Quase sempre □ Geralmente □ Às vezes □ Raramente ou Nunca □
**11. Se você usa protetores auditivos no trabalho, é porque: (por favor, marque todas aquelas que se aplicam):**
A. Seu chefe diz para você fazer
B. Você está fazendo um trabalho ruidoso (ex.: trabalhando em máquinas ruidosas, com estrondos, pancadas, marteladas, etc.)
C. Outros trabalhadores estão fazendo tarefas ruidosas (ex.: trabalhando em máquinas ruidosas, com estrondos, pancadas, marteladas, etc.)
D. Você quer proteger sua audição
E. Você fica incomodado com o ruído
F. Você quer que sua audição esteja preservada para viver com qualidade junto à sua família
G. Seus colegas de trabalho te lembram de usá-los
H. São regras da sua empresa
I. Você recebeu treinamento para usá-los
Outro, por favor, especifique: ___________________________________________
**12. Se você não usa protetores auditivos quando está exposto ao ruído, é porque: (por favor, marque todas aquelas que se aplicam):**
A. Não está claro para você quando você deveria usá-los
B. Você não consegue ouvir adequadamente para fazer seu trabalho (ex. Sinais de aviso, performance de máquinas)
C. Você não consegue se comunicar adequadamente com outros trabalhadores
D. Eles são desconfortáveis
E. Eles atrapalham o uso de outros equipamentos de segurança
F. Você está acostumado com o ruído no trabalho
G. Seus colegas frequentemente não o usam
H. Seus colegas acham engraçado quando você os usa
I. Outras pessoas também fazem tarefas ruidosas sem aviso
J. Outros, por favor, especifique:____________________________________________
**13. Perda auditiva pode ser curada com o uso de aparelhos auditivos (Selecione apenas um);**
**Verdadeiro □ Falso □ Não tenho certeza □**
**14. Medidas de som de _______________ e acima podem prejudicar a audição humana. (selecione apenas uma**):
65 decibels (dBA) □ 70 decibels (dBA) □
85 decibels (dBA) □ Nenhuma destas alternativas □
**15. Sons que são muito altos podem prejudicar __________________, causando perda auditiva (assinale a alternativa que melhor preenche a frase acima e selecione uma apenas):**
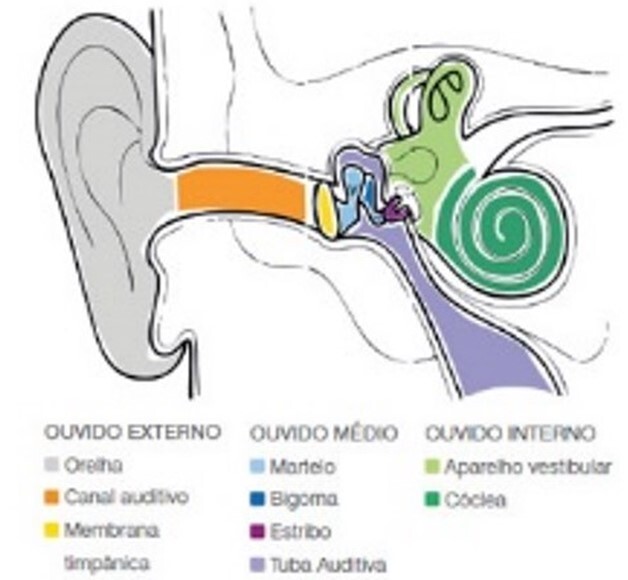
Canal auditivo □ Tímpano ou membrana timpânica □
Células ciliadas da cóclea □ Todas as opções acima □
**16. Perda auditiva causada por sons altos/ruídos é algo que pessoas ___________________ podem ter (assinale a alternativa que melhor preenche a frase acima e selecione apenas uma):**
Acima dos 60 anos □ Acima dos 40 anos □
Acima dos 50 anos □ Em qualquer idade □
**17. Posso proteger a minha audição de ruídos no trabalho usando protetores auditivos. (selecione apenas uma):**
A. A todo momento quando está ruidoso
B. Apenas quando outras pessoas estão usando os seus
C. Apenas quando o ruído me incomoda
D. Apenas quando estou fazendo um trabalho ruidoso
E. Apenas quando meu chefe me orienta a usar
**18. Adquirir uma perda auditiva não é um grande problema para mim (selecione apenas uma):**
Concordo □ Discordo □ Não tenho certeza □
**19. Trabalhadores que estão expostos a sons altos/ruídos o tempo todo não parecem ter perda auditiva, assim, eu não tenho que me preocupar se tiver perda auditiva (selecione apenas uma);**
Concordo □ Discordo □ Não tenho certeza □
**20. Se o ambiente de trabalho estiver ruidoso, e meus colegas de trabalho não estiverem usando protetores auditivos (selecione apenas uma):**
A. Eu prossigo com meu trabalho e os deixo faz erem o que eles quiserem
B. Eu lembro e incentivo meus colegas a usarem seus protetores auditivos
C. Eu também tiro os meus porque eles não estão usando os deles
**21. Durante a semana passada, eu estive exposto a ruídos no trabalho sem usar proteção auditiva (selecione apenas uma):**
Sim □ Não □
**22. Uso protetores auditivos q uando outros trabalhadores estão fazendo um serviço ruidoso no trabalho.**
Sempre □ Quase sempre □ Geralmente □ Às vezes □ Raramente ou Nunca □
**23. Com que frequência seus colegas de trabalho usam protetores auditivos quando o ambiente de trabalho está ruidoso? (Selecione apenas uma):**
Sempre □ Quase sempre □ Geralmente □ Às vezes □ Raramente ou Nunca


Note: For analysis purposes, the correct answers are shown in red in Chart 2, and there may be more than one correct answer in questions 11 and 12

Chart 2 shows in red the answers considered correct for the questionnaire.

### Results of the fifth stage

To verify the reliability of the translated instrument, the split-half method was used, and the sample was divided into two groups: one with 254 employees and the other with 255 employees, totaling 509 participants. One was the upper half and the other was the lower half. Then, we compared the results for each question through the modified C Contingency Coefficient because all questions were nominal questions. Table 2 shows the results.

**Table 2:** Verification of reliability through the Contingency Coefficient (n=509)

QUESTION	CHI-SQUARE	C	p
			
7	0.01	0.0046	0.9301
8	0.45	0.0309	0.5044
10	3.03	0.0775	0.5528
11	4.88	0.0514	0.8446
12	4.98	0.0917	0.8360
			
13	2.78	0.0753	0.2491
14	7.14	0.1189	0.1287
15	2.90	0.0762	0.4073
16	1.42	0.0538	0.7009
17	0.20	0.0205	0.9953
18	1.02	0.0453	0.6005
			
19	0.79	0.0400	0.6237
20	1.05	0.0475	0.5916
21	0.00	0.0000	1.0000
22	4.07	0.0911	0.3966
23	4.03	0.0906	0.4002

Considering a significance level of 0.05 (5%), we found that in all questions p > 0.05, that is, the difference in results between the two groups is not significant, showing independence of results in relation to groups, hence their internal consistency. This result is an indicator of reliability.

## DISCUSSION

Until now, there was no specific questionnaire for the Dangerous Decibels program for workers translated and culturally adapted and validated for Brazilian Portuguese capable of identifying the influences of hearing protection behavior on different scales (barrier and supports), as well as knowledge, habits, and behavior of workers in face of noise in the work environment. It could be used within a hearing preservation program to assess, for example, an educational action on hearing protection. The HPA was developed and validated in English and its effectiveness has been demonstrated^(1,15)^.There were only questionnaires from the Dangerous decibel program for children or adolescents^(15,17,22,23)^.

This study followed the guidelines of the World Health Organization, namely: translation from English into Portuguese, back translation from Portuguese into English, panel of experts, pre-test and interviews and, finally, preparation of the final adjusted version^(18)^.

For cross-cultural validation^(19)^, Table 1 shows content validity and Table 2 shows internal consistency. The understanding of the questions was satisfactory, because in addition to the workers not having difficulties filling out the questionnaire, the correlations were significant, indicating the validity of construction and content for its use.

Therefore, the analysis of the Brazilian Portuguese version of the questionnaire Hearing Protection Assessment Questionnaire (HPA), prepared by Reddy et al.^(15)^, reveals that this is a valid and reproducible instrument to identify and measure the influences of behavior of hearing protection at different scales (barriers and supports) and the identification of knowledge, habits and behavior of Brazilian workers in face of exposure to occupational noise.

### Limitations

The translation, adaptation, and cross-cultural validation of the questionnaire HPA was carried out using a sample of southern Brazilian workers from a meatpacking company, requiring its application in other regions of Brazil.

### Further studies

It is suggested to perform a factor analysis and demonstrate the psychometric properties of the instrument in Brazil to confirm that the instrument, in its current format, is valid, sensitive, and specific for the purpose for which it is intended for Brazilian workers.

In the future, the HPA may be used by health and safety at work teams within the Hearing Preservation Program.

## CONCLUSION

This study resulted in the translation, transcultural adaptation, and validation of the Hearing Protection Assessment Questionnaire (HPA) to be used to assess the use of individual HPD in the occupational field, called Hearing Protection Assessment Questionnaire (HPA).
