# Supplementary material for: Translation, adaptation, and cross-cultural validation into Brazilian portuguese of the hearing protection assessment questionnaire (HPA)
Source: Codas. 2023 May 1;35(2):e20210201. doi: 10.1590/2317-1782/20232021201en (PMC10162647; doi:10.1590/2317-1782/20232021201en)
Supplement: Translation

# Translation: Tradução, adaptação e validação transcultural para o português brasileiro do questionário de Avaliação da Proteção Auditiva (APA)

**DOI:** 10.1590/2317-1782/20232021201pt

**Authors:** Luciana Bramati, Claudia Giglio de Oliveira Gonçalves, Jair Mendes Marques, Ravi Reddy, David Welch, Adriana Bender de Moreira Lacerda

**Affiliations:** 1 Programa de Mestrado e Doutorado em Distúrbios da Comunicação, Universidade Tuiuti do Paraná - UTP - Curitiba (PR), Brasil.; 2 Doctoral Program, School of Health Sciences, Massey University - Auckland, New Zealand.; 3 Department of Computer Schience, University of Auckland - Auckland, New Zealand.; 4 École d’orthophonie et d’audiologie, Université de Montréal - UdeM - Montréal, Canada.

**Keywords:** Audição, Perda Auditiva Induzida pelo Ruído, Dispositivos de Proteção das Orelhas, Comportamento de Risco, Inquéritos e Questionários

## Abstract

**Objetivo:**

O objetivo do presente estudo foi traduzir, adaptar e validar transculturalmente a versão para língua portuguesa brasileira do questionário Hearing Protection Assessment Questionnaire (HPA).

**Método:**

O instrumento original, desenvolvido na língua inglesa, buscou avaliar as barreiras e suportes relacionados ao uso dos dispositivos de proteção auditiva (DPA), assim como o conhecimento, hábitos e atitudes dos trabalhadores frente ao ruído ocupacional. A tradução, adaptação e validação transcultural do questionário foi composta de cinco etapas: Tradução do questionário do inglês para o português; 2) Processo inverso de tradução do português para o inglês; 3) Análise do instrumento por três especialistas na área; 4) Pré-teste do questionário com 10 trabalhadores; 5) Aplicação do instrumento em 509 trabalhadores de uma indústria frigorífica após o exame admissional.

**Resultados:**

os resultados indicam a validade de construção e conteúdo da versão em Português Brasileiro para o seu uso com uma população trabalhadora e a consistência interna do mesmo.

**Conclusão:**

Este estudo resultou na tradução, na adaptação cultural e validação do questionário Hearing Protection Assessment Questionnaire (HPA) com a finalidade de ser usado para avaliar o uso de proteção auditiva individual em âmbito ocupacional, denominado Questionário de Avaliação da Proteção Auditiva (APA).

## INTRODUÇÃO

A identificação do conhecimento, dos hábitos e das atitudes dos trabalhadores a respeito da exposição ao ruído no ambiente de trabalho é recomendada visando a implementação de ações educativas apropriadas. Este cenário sugere a necessidade da utilização de um instrumento específico que realize esta função.

O uso de um instrumento para avaliar a efetividade das intervenções educativas voltadas aos trabalhadores sobre diferentes dimensões ou aspectos relacionados ao ruído ocupacional, aos dispositivos de proteção auditiva (DPA) e a prevenção da perda auditiva induzida por níveis de pressão sonora elevados (PAINPSE) é um importante recurso a ser utilizado pelas equipes de saúde e segurança no trabalho no processo de investigação da exposição à níveis de pressão sonora elevados. Assim, quando se conhece as necessidades dos indivíduos diante do ruído, poderemos ter condições de implementar um processo educativo dentro do Programa de Preservação Auditiva^(1)^.

Instrumentos com esta finalidade foram usados em estudos nacionais e internacionais. Estudos, utilizaram questionários de múltipla escolha, abordando os temas explorados nas intervenções educativas e aplicados pré e pós-intervenção, visando avaliar a efetividade destas intervenções no que se refere ao ruído ocupacional^(2-7)^.

O questionário intitulado “Crenças e Atitudes sobre Proteção Auditiva”, originário da NIOSH^(8,9)^ nos estados Unidos, foi traduzido, adaptado e validado para a língua portuguesa em 2008^(10)^ e usado também em um estudo realizado em 2008^(11)^. Esse questionário é composto por duas partes (A e B) e avalia as crenças e atitudes dos trabalhadores sobre a prevenção da perda auditiva e como eles utilizam os DPA.

Em um estudo realizado com bombeiros, questionários com foco no conhecimento e atitudes frente à PAINPS foram enviados por e-mail pré e pós-intervenção, comprovando através dos resultados que a intervenção educativa mostrou-se eficaz no aumento do conhecimento acerca da PAINPSE, bem como de atitudes positivas quanto ao uso dos DPA, tornando mais frequente o seu uso entre o grupo do estudo (80% após a intervenção, contra 20% antes)^(12)^.

Um estudo publicado em 2018, teve como objetivo descrever o conhecimento dos funcionários sobre a importância do uso dos DPA, os benefícios que trazem a sua utilização e os malefícios decorrentes do não uso constante. As autoras utilizaram questionário elaborado pelas mesmas, aplicados pré e pós intervenção. Os questionários continham 20 questões objetivas, abordando sobre a utilização dos DPA, questões sobre a exposição contínua ao ruído e o conhecimento dos funcionários sobre o tipo de equipamento usado na empresa^(13)^.

Outro estudo realizado em 2013^(14)^, buscou analisar o conforto dos DPA individual como parte de uma intervenção para prevenção de perdas auditivas em trabalhadores expostos a elevados níveis de ruído, por meio da utilização de um questionário de avaliação de conforto.

Desta forma, o uso de questionários como instrumentos de avaliação da efetividade de ações de intervenções educativas é de extrema importância e relevância, fornecendo subsídios valiosos para o direcionamento de ações voltadas à proteção auditiva.

A intervenção educativa do programa Dangerous Decibels adaptado para trabalhadores sugere o uso do instrumento: Hearing Protection Assessment Questionnaire (HPA) desenvolvido por Reddy et al.^(15)^, antes e após a intervenção. Este instrumento avalia as barreiras e os suportes relacionados ao uso dos DPA individual^(16)^, assim como o conhecimento, os hábitos e as atitudes dos trabalhadores frente ao ruído ocupacional. Por não haver até o momento uma versão adaptada para o português brasileiro do HPA que poderia ser usada na intervenção do programa Dangerous Decibels Brasil (DDB) para trabalhadores, optou-se então por realizar a tradução, a adaptação e a validação transcultural do instrumento.

No estudo de Reddy et al.^(15)^, o objetivo foi compreender os fatores pessoais e ambientais que influenciam o comportamento de proteção auditiva em trabalhadores e desenvolver uma intervenção para promovê-lo. O referencial teórico usado para este estudo foi o Modelo Ecológico de Promoção da Saúde; um modelo de planejamento que ajuda a identificar e direcionar influências comportamentais em vários níveis do ambiente social, através de entrevistas semi-estruturadas. A intervenção usada foi o programa Dangerous Decibels adaptado para trabalhadores. O questionário Hearing Protection Assessment Questionnaire (HPA) utilizado por Reddy et al.^(15)^, foi uma ferramenta confiável e válida para identificar as influências do comportamento da proteção auditiva em diferentes níveis^(17)^.

Neste contexto, o objetivo do presente estudo foi traduzir, adaptar e validar transculturalmente para a língua portuguesa, o questionário Hearing Protection Assessment Questionnaire (HPA), desenvolvido por Reddy et al.^(15)^.

## MÉTODO

O presente estudo foi aprovado pelo Comitê de Ética da Universidade Tuiuti do Paraná, processo 2.725.935, e aprovado pela empresa cujos funcionários participaram desta pesquisa. Cabe salientar que todos os indivíduos envolvidos assinaram o Termo de Consentimento Livre e Esclarecido.

### Instrumento

O questionário de avaliação da proteção auditiva que avalia cinco escalas (APA), desenvolvido e descrito por Reddy^(1)^ avalia barreiras e suportes, conhecimentos, atitudes e comportamentos em relação aos DPA^(1,15)^.

O conhecimento, atitudes e comportamentos foram adaptados de um questionário usado para avaliar a eficácia do programa Dangerous Decibels^(17)^. As escalas relacionadas ao conhecimento, atitudes e comportamento apresentam perguntas de múltipla escolha, cada uma das quais com apenas uma resposta correta. Existem cinco questões para a escala de conhecimento sobre a ciência do som, perda auditiva e conservação auditiva (questões de 13 a 17), duas questões relacionadas à medida de atitudes sobre proteção contra o ruído e proteção auditiva (questões 18 e 19), duas perguntas sobre atitudes de comportamento de segurança no trabalho (questões 7 e 8) e três perguntas sobre comportamento (questões 10, 20 e 21).

As questões relacionadas às barreiras e os suportes, descrevem os motivos pelos quais os trabalhadores usavam (suportes) ou não usavam (barreiras) os protetores auditivos quando expostos ao ruído no trabalho. As duas questões relacionadas ao Suporte são as questões 9 e 11. A questão 11 apresenta quatro subescalas nas respostas (cultura de segurança, reconhecimento do risco, motivação de comportamento e cultura de segurança). A questão relacionada as Barreiras - é a questão 12, com duas subescalas nas respostas (justificativa do risco e restrições ao uso DPA).

O questionário também inclui itens demográficos, como sexo e idade (questões 1 a 6); um item para identificar a frequência autorreferida do uso individual de DPA (questão 22) e um item para identificar a frequência autorreferida do comportamento de proteção auditiva dos colegas de trabalho (questão 23).

O questionário deve ser analisado comparando as respostas de cada questão das cinco dimensões separadamente (atitude, comportamento, conhecimento, suportes e barreiras) no pré e no pós-intervenção educativa, visando detectar diferenças de resultados entre os dois momentos.

Como as cinco dimensões avaliadas no questionário pré e pós-intervenção educativa, apresentam números diferentes de itens, as pontuações de respostas corretas devem ser convertidas em porcentagens para permitir a comparabilidade entre elas.

### Tradução, adaptação e validação transcultural do instrumento

O processo de tradução, adaptação e validação transcultural foi constituído por cinco etapas de acordo com as recomendações da OMS^(18)^ e da COSMIN^(19)^. A participação do pesquisador na adaptação de um instrumento é desejável, uma vez que permite citar os conceitos explorados, reformular as questões e evitar as locuções e expressões idiomáticas^(20)^.

*A primeira etapa* foi a tradução do questionário do inglês para o português, realizada por um professor bilíngue e revisada por dois especialistas da área. Nessa etapa foram avaliadas a equivalência semântica (gramática e vocabulário) e a equivalência cultural de cada item (experiências vivenciadas dentro do contexto cultural da sociedade).

*A segunda etapa,* foi redigir a versão final com os ajustes realizada por um especialista da área, e posteriormente, encaminhada para um segundo especialista (sem qualquer contato ou informação sobre a versão original), para que este procedesse ao processo inverso de tradução do português para o inglês, sendo revisada e comparada com a versão original por três especialistas (bilíngues) para verificação se houve qualquer descaracterização do questionário.

*Na terceira etapa*, o questionário foi enviado a três especialistas na área, juntamente com um instrumento para que as mesmas pudessem expor seus comentários frente à tradução realizada.

Este método procura facilitar o entendimento e tornar o instrumento aplicável ao idioma nativo do português brasileiro, mas mantendo a equivalência entre o original e a tradução.

*Na quarta etapa*, foram selecionados dez trabalhadores (aleatoriamente) de outra empresa para integrarem o pré-teste do questionário, em horário que não interferisse em suas atividades laborais.

Os questionários do pré-teste foram aplicados pela pesquisadora em horário pré-determinado e, após a leitura e explicação do aplicador (sobre os objetivos da pesquisa e como deveriam ser efetuadas as respostas) foi solicitado aos trabalhadores que respondessem o questionário, registrassem as dificuldades de interpretação, opinassem sobre a linguagem utilizada (se estava adequada e/ou havia palavra ou expressão desconhecida), bem como, indicassem as dificuldades encontradas em respondê-lo. Também foi avaliado o cuidado específico com as instruções de preenchimento e a coerência da apresentação. Nesta etapa não foi necessária nenhuma mudança no questionário, pois os trabalhadores não apresentaram dificuldades de interpretação e aprovaram a linguagem utilizada.

*Na quinta etapa* foi realizada a aplicação do questionário por uma Técnica de Segurança do Trabalho, responsável pelos grupos de integração (momento de admissão do trabalhador na empresa). Participaram desta etapa trabalhadores de uma indústria frigorífica do município de Chapecó, estado de Santa Catarina - Brasil. Como critério de inclusão, participaram os trabalhadores alfabetizados, maiores de 18 anos, de ambos os sexos, que foram admitidos na empresa no período de setembro/2018 a março/2019 e como critério de exclusão, os trabalhadores admitidos no mesmo período, que não sabiam ler ou escrever, que não dominavam a língua portuguesa e os menores de 18 anos.

### Análise estatística

Para a análise da validade de conteúdo foi utilizado o IVC (índice de validade de conteúdo), que é calculado com base nas avaliações dos juízes (especialistas)^(8)^. O IVC avalia a proporção ou porcentagem de concordância de especialistas sobre determinados aspectos de um instrumento e de seus itens^(21)^.

A confiabilidade do instrumento traduzido foi realizada através do método da divisão em metade (*split-half method*), sendo a amostra dividida em dois grupos, um de 254 funcionários e outro de 255 funcionários, sendo um, a metade superior e outro a metade inferior, e em seguida comparamos os resultados, para cada questão, através do Coeficiente de Contingência modificado C, para o caso de questões nominais, verificando sua significância. O nível de significância utilizado foi 0,05 (5%).

## RESULTADOS

### Resultados da primeira à quarta etapa

O Quadro 1 um apresenta a versão original do questionário, o processo de **Tradução, retro tradução e adaptação** das questões e das opções de resposta.

**Quadro 1:** Versão original do questionário, o processo de **Tradução, retro tradução e adaptação** das questões e das opções de resposta.

SCALE	ORIGINAL ENGLISH VERSION		TRANSLATION INTO BRAZILIAN PORTUGUESE		BACK-TRANSLATION INTO ENGLISH		SPECIALISTS COMMITTEE: SEMANTIC, LANGUAGE, CULTURAL, AND LINGUISTIC EQUIVALENCE	
Attitude	7. Please read the two statements carefully and choose the one which is most true for you: Please choose either A or B		7. Por favor, leia as duas frases cuidadosamente e escolha aquela que é mais verdadeira para você: (escolha A ou B)		7. Please read the two statements carefully and choose the one that is most true to you: (please choose A or B)		7. Por favor, leia as duas frases cuidadosamente e escolha aquela que é mais verdadeira para você: Por favor, escolha A ou B	
	A. Safety is at the forefront of my mind when working		A. Segurança está em primeiro lugar em minha mente quando trabalho		A. Safety comes first in my mind when I work		A. Para mim, a segurança está em primeiro lugar quando eu trabalho.	
	B. Safety is important, but other factors sometimes limit my ability to work safely		B. Segurança é importante, mas outros fatores às vezes limitam minha habilidade para trabalhar de forma segura		B. Security is important, however other factors sometimes limit my ability to work safely		B. Para mim, a segurança é importante, mas outros fatores ou condições de trabalho, às vezes limitam à minha maneira de trabalhar de forma segura.	
Attitude	8. Please read the two statements carefully and choose the one which is most true for you: Please choose either A or B		8. Por favor, leia as duas frases cuidadosamente e escolha aquela que é mais verdadeira para você: (escolha A ou B)		8. Please read the two statements carefully and choose the one that is most true to you: (please choose A or B)		8. Por favor, leia as duas frases cuidadosamente e escolha aquela que é mais verdadeira para você:	
	A. Injuries occur at work because people don’t take enough interest in safety		A. Os danos ocorrem no trabalho porque as pessoas não têm interesse suficiente em segurança		A. Damage occurs at work because people do not have enough interest in safety		A. Os danos ocorrem no trabalho porque as pessoas não têm interesse suficiente em segurança	
	B. Injuries at work will always occur, no matter how hard people try to prevent them		B. Os danos no trabalho sempre ocorrerão, não importa o quanto as pessoas tentem preveni-los(as)		B. Damage at work will always occur, no matter how much people try to prevent it.		B. Os danos sempre ocorrerão no trabalho, não importa o quanto as pessoas tentem preveni-los(as)	
Support	9. I have earplugs and or earmuffs to use at work		9. Eu tenho protetores auditivos para usar no meu trabalho		9. I have hearing protectors to use in my work		9. Eu recebo protetores auditivos para usar no meu trabalho	
	□ Yes	□ No	Sim □	Não □	□ Yes	□ No	Sim □	Não □
Behavior	10. I wear earplugs and or earmuffs when it is noisy at work (please circle one)		10. Eu uso só protetores auditivos quando tem barulho no meu ambiente de trabalho (por favor, assinale uma das opções abaixo)		10. I wear hearing protectors when it is noisy at work (please check one of the options below)		10. Eu uso protetores auditivos quando tem ruído no trabalho (por favor, assinale uma das opções abaixo)	
	□Always		Sempre □		□Always		Sempre □	
	□Almost Always		Quase sempre □		□Almost Always		Quase sempre □	
	□Usually		Geralmente □		□Usually		Geralmente □	
	□Often		Muitas vezes □		□Often		Muitas vezes □	
	□Sometimes		Às vezes □		□Sometimes		Às vezes □	
	□Rarely or Never		Raramente ou Nunca □		□Rarely or Never		Raramente ou Nunca □	
Support/ subscales	11. If you wear earmuffs or earplugs when exposed to noise, it is because: (please tick all those that apply)		11. Se você usa protetores auditivos no trabalho, é porque: (marque todas aquelas que se aplicam)		11. If you wear hearing protectors at work, it is because: (check all that apply)		11. Se você usa protetores auditivos no trabalho, é porque: (por favor, marque todas aquelas que se aplicam)	
Safety culture	A. Your boss tells you to		A. Seu chefe diz para você fazer		A. Your boss tells you to do		A. Seu chefe diz para você usar	
Hazard recognition	B. You are doing a noisy job (e.g., working on noisy machine, banging, hammering, etc.)		B. Você está fazendo um trabalho barulhento (ex.: trabalhando em máquinas ruidosas, com estrondos, pancadas, marteladas, etc.)		B. You are doing a noisy work (e.g., working on noisy machines, with bangs, banging, hammering, etc.)		B. Você está fazendo um trabalho ruidoso (ex.: trabalhando em máquinas ruidosas, com estrondos, pancadas, marteladas, etc.)	
Hazard recognition	C. Other workers are doing noisy jobs (e.g., working on noisy machine, banging, hammering, etc.)		C. Outros trabalhadores estão executando tarefas barulhentas (ex.: trabalhando em máquinas ruidosas, com estrondos, pancadas, marteladas, etc.)		C. Other workers are performing noisy tasks (e.g., working on noisy machines, with bangs, bangs, hammering, etc.)		C. Outros trabalhadores estão fazendo tarefas ruidosas (ex.: trabalhando em máquinas ruidosas, com estrondos, pancadas, marteladas, etc.)	
Behavior motivation	D. You want to protect your hearing		D. Você quer proteger sua audição		D. You want to protect your hearing		D. Você quer proteger sua audição	
Behavior motivation	E. You are annoyed by the noise		E. Você fica chateado com o barulho		E. You are upset / annoyed by the noise		E. Você fica incomodado com o ruído	
Behavior motivation	F. You want your hearing to be good to live a good life with your family		F. Você quer que sua audição esteja boa para viver uma vida com qualidade com sua família		F. You want a good hearing to have a good live and quality of life with your family		F. Você quer que sua audição esteja preservada para viver com qualidade junto à sua família	
Behavior motivation	G. Your workmates remind you to wear them		G. Seus colegas de trabalho te lembram de usá-los		G. Your co-workers remind you to use them		G. Seus colegas de trabalho te lembram de usá-los	
Safety culture	H. It is your company rules		H. São regras de sua empesa		H. It is your Company rules		H. São regras da sua empresa	
Safety culture	I. You have received training to wear them		I. Você recebeu treinamento para usá-los		I. You have received training to use them		I. Você recebeu treinamento para usá-los	
	J. Other, please specify:		J. Outro, por favor, especifique:		J. Other, please specify:		J. Outro, por favor, especifique: _______________________________	
Barriers/ subscales	12. If you don’t wear earmuffs or earplugs when exposed to noise, it is because: (please tick all those that apply)		12. Se você não usa protetores auditivos quando está exposto ao barulho, é porque: (marque todas aquelas que se aplicam)		12. If you don’t use hearing protectors when exposed to noise, it is because: (check all that apply)		12. Se você não usa protetores auditivos quando está exposto ao ruído, é porque: (por favor, marque todas aquelas que se aplicam)	
Risk justification	A. You are not clear as to when you should wear them		A. Não está claro para você quando você deveria usá-los		A. It is not clear to you when you should use them		A. Não está claro para você quando você deveria usá-los	
Hpd constraints	B. You can’t hear properly to do your work (e.g., warning signals, machine performance)		B. Você não consegue ouvir apropriadamente para fazer seu trabalho (ex. Sinais de aviso, performance de máquinas)		B. You cannot hear properly to do your job (e.g., warning signs, machine performance)		B. Você não consegue ouvir adequadamente para fazer seu trabalho (ex. Sinais de aviso, performance de máquinas)	
Hpd constraints	C. You can’t communicate properly with other workers		C. Você não consegue se comunicar apropriadamente com outros trabalhadores		C. You can’t to communicate properly with other workers		C. Você não consegue se comunicar adequadamente com outros trabalhadores	
Hpd constraints	D. They are uncomfortable		D. Eles são desconfortáveis		D. They are uncomfortable		D. Eles são desconfortáveis	
Hpd constraints	E. They get in the way of other safety equipment		E. Eles atrapalham o uso de outros equipamentos de segurança		E. They get in the use of other safety equipment		E. Eles atrapalham o uso de outros equipamentos de segurança	
Risk justification	F. You are used to noise at work		F. Você está acostumado com barulho no trabalho		F. You just used to noise at work		F. Você está acostumado com o ruído no trabalho	
Risk justification	G. Your co‐workers often don’t wear them		G. Seus colegas frequentemente não o usam		G. Your colleagues often don't use it		G. Seus colegas frequentemente não o usam	
Risk justification	H. Your co‐workers find it funny when you wear them		H. Seus colegas acham engraçado quando você os usa		H. Your colleagues find it funny when you use them		H. Seus colegas acham engraçado quando você os usa	
Risk justification	I. Someone else does something noisy without warning		I. Outras pessoas também fazem tarefas barulhentas sem aviso		I. Someone else does something noisy tasks without warning		I. Outras pessoas também fazem tarefas ruidosas sem avisar	
	J. Other, please specify:		J. Outros, por favor, especifique:		J. Other, please specify:		J. Outros, por favor, especifique:	
Knowledge	13. Hearing loss can be cured by getting hearing aids. (tick only one)		13. A Perda auditiva pode ser curada por aparelhos auditivos (Selecione apenas um)		13. Hearing loss can be cured by hearing aids (Select only one)		13. Perda auditiva pode ser curada com o uso de aparelhos auditivos (Selecione apenas um)	
	□True		Verdadeiro □		□True		Verdadeiro □	
	□False		Falso □		□False		Falso □	
	□Not sure		Não tenho certeza □		□Not sure		Não tenho certeza □	
Knowledge	14. Sounds measuring _______ and over are damaging to human hearing. (tick only one)		14. Medidas de som de _______________ e acima podem prejudicar a audição humana. (selecione apenas uma)		14. Sound measurements of _______________ and over are damaging to human hearing. (select only one)		14. Medidas de som de _______________ e acima podem prejudicar a audição humana. (selecione apenas uma)	
	65 decibels (dBA) □		65 decibels (dBA) □		65 decibels (dBA) □		65 decibels (dBA) □	
	70 decibels (dBA) □		70 decibels (dBA) □		70 decibels (dBA) □		70 decibels (dBA) □	
	85 decibels (dBA) □		85 decibels (dBA) □		85 decibels (dBA) □		85 decibels (dBA) □	
	Not sure □		Nenhuma destas alternativas □		None of these alternatives □		Nenhuma destas alternativas □	
Knowledge	15. Sounds that are too loud can damage the _______, causing hearing loss. (tick only one)		15. Os sons muito altos podem prejudicar __________________, causando perda auditiva (selecione uma apenas)		15. Sounds that are too loud can damage the __________________, causing hearing loss (select only one)		15. Sons muito altos podem prejudicar __________________, causando perda auditiva (assinale a alternativa que melhor preenche a frase acima e selecione uma apenas)	

	Ear drum □		Tímpano □		Ear drum □		Canal Auditivo □	
	Ear canal □		Canal Auditivo □		Ear canal □		Tímpano ou membrana timpanica □	
	Hair cells of the inner ear □		Células ciliadas do Ouvido Interno □		Hair cells of the inner ear □		Células ciliadas da cóclea □	
	All of the above □		Todas as opções acima □		All of the above □		Todas as opções acima □	
Knowledge	16. Hearing loss caused by loud sounds is something people ________ may have. (tick only one)		16. A perda auditiva causada por sons altos é algo que pessoas ___________________ podem ter (selecione apenas uma)		16. Hearing loss caused by loud sounds is something people ___________________ may have (select only one)		16. Perda auditiva causada por sons altos/ruídos é algo que pessoas ___________________ podem ter (assinale a alternativa que melhor preenche a frase acima e selecione apenas uma)	
	Over age 60 □		Acima dos 60 anos □		Over age 60 □		Acima dos 60 anos □	
	Over age 50 □		Acima dos 50 anos □		Over age 50 □		Acima dos 50 anos □	
	Over age 40 □		Acima dos 40 anos □		Over age 40 □		Acima dos 40 anos □	
	At any age □		Em qualquer idade □		At any age □		Em qualquer idade □	
Knowledge	17. I can protect my hearing from noise at work by wearing earmuffs or earplugs (tick only one)		17. Posso proteger minha audição de barulhos no trabalho usando protetores auditivos. (selecione apenas uma)		17. I can protect my hearing from noise at work by using hearing protection. (select only one)		17. Posso proteger a minha audição do ruido no trabalho usando protetores auditivos. (selecione apenas uma)	
	All the time when it is noisy □		Todo momento quando está barulhento □		All the time when it is noisy □		A Todo momento quando está ruidoso □	
	Only when I am doing a noisy job □		Apenas quando outras pessoas estão usando os seus □		Only when I am doing a noisy job □		Apenas quando outras pessoas estão usando os seus □	
	Only when others are wearing theirs □		Apenas quando o barulho me incomoda □		Only when others are wearing theirs □		Apenas quando o ruído me incomoda □	
	Only when my boss tells me to □		Apenas quando estou fazendo um trabalho barulhento □		Only when my boss tells me to □		Apenas quando estou fazendo um trabalho ruidoso □	
	Only when the noise annoys me □		Apenas quando meu chefe me orienta a usar □		Only when the noise annoys me □		Apenas quando meu chefe me orienta a usar □	
Attitude	18. Having a hearing loss is not a big deal (tick only one)		18. Ter perda auditiva não é um grande problema (selecione apenas uma)		18. Having hearing loss is not a big problem (select only one)		18. Adquirir uma perda auditiva não é um grande problema para mim (selecione apenas uma)	
	Agree □		Concordo □		Agree □		Concordo □	
	Disagree □		Discordo □		Disagree □		Discordo □	
	Not sure □		Não tenho certeza □		I'm not sure □		Não tenho certeza □	
Attitude	19. Workers who listen to loud sounds all the time don't seem to have a hearing loss, so I don't have to worry about getting a hearing loss. (tick only one)		19. Trabalhadores que escutam sons altos o tempo todo não parecem ter perda auditiva, assim, eu não tenho que me preocupar se tiver perda auditiva (selecione uma apenas)		19. Workers who listen loud sounds all the time don't seem to have a hearing loss, so I don't have to worry about getting a hearing loss (select only one)		19. Trabalhadores que estão expostos a ruídos o tempo todo não parecem ter perda auditiva, assim, eu não tenho que me preocupar se tiver perda auditiva (selecione uma apenas)	
	Agree □		Concordo □		Agree □		Concordo □	
	Disagree □		Discordo □		Disagree □		Discordo □	
	Not sure □		Não tenho certeza □		I'm not sure □		Não tenho certeza □	
Behavior	20. If it is noisy, and my workmates are not wearing earmuffs or earplugs. (tick only one)		20. Se estiver barulhento, e meus colegas de trabalho não estiverem usando protetores auditivos (selecione uma apenas)		20. If it is noisy, and my co-workers are not wearing hearing protection (select only one)		20. Se o ambiente de trabalho estiver ruidoso, e meus colegas de trabalho não estiverem usando protetores auditivos (selecione uma apenas)	
	I carry on with my work and let them do what they want □		Eu prossigo com meu trabalho e os deixo fazerem o que eles quiserem □		I carry on with my work and let them do what they want □		Eu prossigo com meu trabalho e os deixo fazerem o que eles quiserem □	
	I remind and encourage them to wear their earplugs or earmuffs □		Eu lembro e incentivo meus colegas a usarem seus protetores auditivos □		I remind and encourage my colleagues to wear their hearing protectors □		Eu lembro e incentivo meus colegas a usarem seus protetores auditivos □	
	I also take mine off because they are not wearing theirs □		Eu também tiro os meus porque eles não estão usando os deles □		I also take mine because they are not using theirs □		Eu também tiro os meus porque eles não estão usando os deles □	
Behavior	21. During the past week, I have been around dangerously loud sounds at work without wearing hearing protection. (tick only one)		21. Durante a semana passada, eu estive em contato com sons altos no trabalho sem usar proteção auditiva. (selecione uma apenas)		21. During the past week, I have been around dangerously loud sounds at work without wearing hearing protection. (select one only)		**21.** Durante a semana passada, eu estive exposto a ruídos no trabalho sem usar proteção auditiva. (selecione uma apenas)	
	Yes □	No □	Sim □	Não □	Yes □	No □	Sim □	Não □
Behavior	22. I wear earplugs or earmuffs when other workers are doing a noisy job at work (tick only one)		22. Uso protetores auditivos quando outros trabalhadores estão fazendo um serviço barulhento no trabalho.		22. I use hearing protection when other workers are doing a noisy job at work.		22. Eu uso protetores auditivos quando outros trabalhadores estão fazendo um serviço ruidoso no trabalho.	
	Always □		Sempre □		Always □		Sempre □	
	Almost always □		Quase sempre □		Almost always □		Quase sempre □	
	Usually □		Geralmente □		Usually □		Geralmente □	
	Often sometimes □		Frequentemente □		Often sometimes □		Frequentemente □	
	Rarely □		Raramente □		Rarely □		Raramente □	
	Never □		Nunca □		Never □		Nunca □	
Behavior	23. How often do your workmates wear earmuffs or earplugs when it is noisy? (tick only one)		23. Com que frequência seus colegas de trabalho usam protetores auditivos quando está barulhento? (Selecione uma apenas)		23. How often do your co-workers wear hearing protection when it is noisy? (Select one only)		23. Com que frequência seus colegas de trabalho usam protetores auditivos quando o ambiente de trabalho está ruidoso? (Selecione uma apenas)	
	Always □		Sempre □		Always □		Sempre □	
	Almost always □		Quase sempre □		Almost always □		Quase sempre □	
	Usually □		Geralmente □		Usually □		Geralmente □	
	Often sometimes □		Às vezes □		Often sometimes □		Às vezes □	
	Rarely □		Raramente □		Rarely □		Raramente □	
	Never □		Nunca □		Never □		Nunca □	

A equipe de especialistas que analisou as traduções (terceira etapa), apontou que houve correspondência nos itens traduzidos, equivalência semântica entre as duas traduções para a maioria das questões e ausência de dificuldades de tradução. Foram realizados ajustes nas diferenças de concordância verbal. A contra tradução com a versão original, não revelou necessidade de alterações em estruturas gramaticais, quando a versão em português foi traduzida para o inglês.

O julgamento do comitê de especialistas revela que as questões 8, 9, 10, 13, 14 e 16 alcançaram consenso entre os três juízes. As questões 11 (B/C/E), 12 (F/I), 17, 19, 20, 22 e 23 foram consideradas itens com necessidade de pequena revisão para serem representativas, e as questões 7, 15, 18 e 21 sofreram mudanças visando facilitar o entendimento na língua portuguesa, considerando as diferenças culturais entre os idiomas.

O Índice de Validade de Conteúdo (IVC) avaliou a proporção de concordância de especialistas sobre o instrumento e seus itens. A proporção de questões que receberam uma pontuação pelos juízes (especialistas) está apresentada na Tabela 1.

**Tabela 1:** Índice de validade de conteúdo (IVC)

QUESTÃO	IVC		QUESTÃO	IVC	QUESTÃO	IVC
Q7	0,33		Q13	1,00	Q19	1,00
Q8	1,00		Q14	1,00	Q20	1,00
Q9	1,00		Q15	0,67	Q21	0,67
Q10	1,00		Q16	1,00	Q22	1,00
Q11	1,00		Q17	1,00	Q23	1,00
Q12	1,00		Q18	0,67		

Na Tabela 1, pode-se verificar que as questões com IVC = 1,00, receberam entre os três juízes escores 3 ou 4, portanto com validade de conteúdo adequada. As questões com IVC = 0,33 ou IVC = 0,67 foram aquelas que receberam pelo menos um escore 1 ou 2, portanto essas questões foram revisadas. A Tabela 1 apresenta Índice de Validade de Conteúdo (IVC).

Na quarta etapa, os dez trabalhadores responderam ao questionário (Quadro 2) eles não apresentaram dificuldades de interpretação e consideraram a linguagem adequada. Após responderem ao questionário, alguns fizeram questionamentos sobre os níveis confortáveis de ruído e tipos de protetores auditivos utilizados.

**Quadro 2:** Versão final do português brasileiro do questionário: **Avaliação de Proteção Auditiva (APA)**

Como responder: preencha a sua resposta com um **X** ou escreva as suas respostas nas linhas.

**1. Gênero:** Masculino □ Feminino □
**2. Idade:** _____________anos Data de Nascimento: _____/_____/_____
**3. Cargo:** _______________________________ Turno: ________________
**4. A qual grupo étnico você pertence?** __________________________________

**5. Qual é seu país de nascimento?**
Brasil □ outro, por favor, especifique: _____________________________
**6. Se você respondeu « outro » para a questão 5, há quantos anos você está no Brasil?** ______
**7. Por favor, leia as duas frases cuidadosamente e escolha aquela que é mais verdadeira para você. Por favor, escolha A ou B:**
A. Para mim, a segurança está em primeiro lugar quando eu trabalho.
B. Para mim, a segurança é importante, mas outros fatores ou condições de trabalho, às vezes limitam à minha maneira de trabalhar de forma segura.
**8. Por favor, leia as duas frases cuidadosamente e escolha aquela que é mais verdadeira para você. Por favor, escolha A ou B:**
A. Os danos ocorrem no trabalho porque as pessoas não têm interesse suficiente em segurança
B. Os danos sempre ocorrerão no trabalho, não importa o quanto as pessoas tentem preveni-los
**9. Eu recebo protetores auditivos disponíveis para usar no meu trabalho.**
Sim □ Não □
**10. Eu uso protetores auditivos quando tem ruído no trabalho (por favor, assinale uma das opções abaixo):**
Sempre □ Quase sempre □ Geralmente □ Às vezes □ Raramente ou Nunca □
**11. Se você usa protetores auditivos no trabalho, é porque: (por favor, marque todas aquelas que se aplicam):**
A. Seu chefe diz para você fazer
B. Você está fazendo um trabalho ruidoso (ex.: trabalhando em máquinas ruidosas, com estrondos, pancadas, marteladas, etc.)
C. Outros trabalhadores estão fazendo tarefas ruidosas (ex.: trabalhando em máquinas ruidosas, com estrondos, pancadas, marteladas, etc.)
D. Você quer proteger sua audição
E. Você fica incomodado com o ruído
F. Você quer que sua audição esteja preservada para viver com qualidade junto à sua família
G. Seus colegas de trabalho te lembram de usá-los
H. São regras da sua empresa
I. Você recebeu treinamento para usá-los
Outro, por favor, especifique: ___________________________________________
**12. Se você não usa protetores auditivos quando está exposto ao ruído, é porque: (por favor, marque todas aquelas que se aplicam):**
A. Não está claro para você quando você deveria usá-los
B. Você não consegue ouvir adequadamente para fazer seu trabalho (ex. Sinais de aviso, performance de máquinas)
C. Você não consegue se comunicar adequadamente com outros trabalhadores
D. Eles são desconfortáveis
E. Eles atrapalham o uso de outros equipamentos de segurança
F. Você está acostumado com o ruído no trabalho
G. Seus colegas frequentemente não o usam
H. Seus colegas acham engraçado quando você os usa
I. Outras pessoas também fazem tarefas ruidosas sem aviso
J. Outros, por favor, especifique:____________________________________________
**13. Perda auditiva pode ser curada com o uso de aparelhos auditivos (Selecione apenas um);**
**Verdadeiro □ Falso □ Não tenho certeza □**
**14. Medidas de som de _______________ e acima podem prejudicar a audição humana. (selecione apenas uma**):
65 decibels (dBA) □ 70 decibels (dBA) □
85 decibels (dBA) □ Nenhuma destas alternativas □
**15. Sons que são muito altos podem prejudicar __________________, causando perda auditiva (assinale a alternativa que melhor preenche a frase acima e selecione uma apenas):**

Canal auditivo □ Tímpano ou membrana timpânica □
Células ciliadas da cóclea □ Todas as opções acima □
**16. Perda auditiva causada por sons altos/ruídos é algo que pessoas ___________________ podem ter (assinale a alternativa que melhor preenche a frase acima e selecione apenas uma):**
Acima dos 60 anos □ Acima dos 40 anos □
Acima dos 50 anos □ Em qualquer idade □
**17. Posso proteger a minha audição de ruídos no trabalho usando protetores auditivos. (selecione apenas uma):**
A. A todo momento quando está ruidoso
B. Apenas quando outras pessoas estão usando os seus
C. Apenas quando o ruído me incomoda
D. Apenas quando estou fazendo um trabalho ruidoso
E. Apenas quando meu chefe me orienta a usar
**18. Adquirir uma perda auditiva não é um grande problema para mim (selecione apenas uma):**
Concordo □ Discordo □ Não tenho certeza □
**19. Trabalhadores que estão expostos a sons altos/ruídos o tempo todo não parecem ter perda auditiva, assim, eu não tenho que me preocupar se tiver perda auditiva (selecione apenas uma);**
Concordo □ Discordo □ Não tenho certeza □
**20. Se o ambiente de trabalho estiver ruidoso, e meus colegas de trabalho não estiverem usando protetores auditivos (selecione apenas uma):**
A. Eu prossigo com meu trabalho e os deixo faz erem o que eles quiserem
B. Eu lembro e incentivo meus colegas a usarem seus protetores auditivos
C. Eu também tiro os meus porque eles não estão usando os deles
**21. Durante a semana passada, eu estive exposto a ruídos no trabalho sem usar proteção auditiva (selecione apenas uma):**
Sim □ Não □
**22. Uso protetores auditivos q uando outros trabalhadores estão fazendo um serviço ruidoso no trabalho.**
Sempre □ Quase sempre □ Geralmente □ Às vezes □ Raramente ou Nunca □
**23. Com que frequência seus colegas de trabalho usam protetores auditivos quando o ambiente de trabalho está ruidoso? (Selecione apenas uma):**
Sempre □ Quase sempre □ Geralmente □ Às vezes □ Raramente ou Nunca


Obs.: Para fins de análise, as respostas corretas estão apresentadas na cor vermelha no Quadro 2, podendo haver mais de uma resposta correta nas questões 11 e 12

As respostas consideradas corretas para o questionário estão apresentadas na cor vermelha, no Quadro 2.

### Resultados da quinta etapa

Para a verificação da confiabilidade do instrumento traduzido foi utilizado o método da divisão em metade (*split-half method*), onde dividimos os 509 participantes em dois grupos, um de 254 e outro de 255 funcionários, sendo um, a metade superior e o outro a metade inferior, e em seguida comparamos os resultados, para cada questão, através do Coeficiente de Contingência modificado C, pois as questões são todas nominais. Os resultados obtidos estão na Tabela 2.

**Tabela 2:** Verificação da confiabilidade através do Coeficiente de Contingência (n=509)

QUESTÃO	QUI-QUADRADO	C	p
			
7	0,01	0,0046	0,9301
8	0,45	0,0309	0,5044
10	3,03	0,0775	0,5528
11	4,88	0,0514	0,8446
12	4,98	0,0917	0,8360
			
13	2,78	0,0753	0,2491
14	7,14	0,1189	0,1287
15	2,90	0,0762	0,4073
16	1,42	0,0538	0,7009
17	0,20	0,0205	0,9953
18	1,02	0,0453	0,6005
			
19	0,79	0,0400	0,6237
20	1,05	0,0475	0,5916
21	0,00	0,0000	1,0000
22	4,07	0,0911	0,3966
23	4,03	0,0906	0,4002

Utilizando o nível de significância de 0,05 (5%) verificamos que em todas as questões p > 0,05, ou seja, a diferença de resultados entre os dois grupos não é significativa, mostrando a independência dos resultados em relação aos grupos, consequentemente a consistência interna deles. Esse resultado é um indicador de confiabilidade.

## DISCUSSÃO

Não existia, até o momento, um questionário específico do programa Dangerous Decibels para trabalhadores, traduzido e culturalmente adaptado e validado para o Português Brasileiro, capaz de identificar as influências do comportamento da proteção auditiva em diferentes escalas (barreira e suportes), assim como os conhecimentos, os hábitos e os comportamentos dos trabalhadores diante do ruído no ambiente de trabalho, que poderia ser usado dentro de um programa de preservação auditiva para se avaliar, por exemplo uma ação educativa sobre proteção auditiva. O APA foi desenvolvido e validado em inglês e sua efetividade foi demonstrada^(1,15)^. Existiam apenas os questionários do programa Dangerous decibel para crianças ou adolescentes^(15,17,22,23)^.

Este estudo seguiu as diretrizes da Organização Mundial da Saúde, sendo elas a tradução do inglês para o português, a retro tradução do português para o inglês, o painel de especialistas, o pré-teste e entrevistas e por fim, a elaboração da versão final ajustada^(18)^.

Para a validação transcultural^(19)^, a validade de conteúdo foi demonstrada na Tabela 1 e a consistência interna foi demonstrada pela Tabela 2. O entendimento das questões foi satisfatório, pois além dos trabalhadores não apresentarem dificuldades na hora do preenchimento do questionário, as correlações foram significativas indicando a validade de construção e conteúdo para o seu uso.

Sendo assim, a análise da versão em português brasileiro do questionário Hearing Protection Assessment Questionnaire (HPA), elaborado por Reddy et al.^(15)^, revela que este é um instrumento válido e reprodutível para identificar e mensurar as influências do comportamento da proteção auditiva em diferentes escalas (barreiras e suportes) e a identificação do conhecimento, dos hábitos e do comportamento dos trabalhadores brasileiros diante da exposição ao ruído ocupacional.

### Limitações

A tradução, adaptação e validação transcultural do questionário denominado APA foi realizada usando uma amostra de trabalhadores sul brasileiros de uma empresa frigorífica, sendo necessário à sua aplicação em outras regiões do país.

### Estudos futuros

Sugere-se a realização de análise fatorial e demonstração das propriedades psicométricas do instrumento no Brasil, para confirmar que o instrumento, em seu formato atual, é válido, sensível e específico para a finalidade a que se destina em trabalhadores brasileiros.

Futuramente o APA poderá ser utilizado pelas equipes de saúde e segurança no trabalho dentro do Programa de Preservação Auditiva.

## CONCLUSÃO

Este estudo resultou na tradução, na adaptação e validação transcultural do questionário Hearing Protection Assessment Questionnaire (HPA) com a finalidade de ser usado para avaliar o uso dos DPA individual em âmbito ocupacional, denominado Questionário de Avaliação da Proteção Auditiva (APA).
